# Bilateral Middle Cerebral Artery Occlusion: A Successful Case of Bilateral Thrombectomy

**DOI:** 10.7759/cureus.48094

**Published:** 2023-11-01

**Authors:** Gordon White, Mariel Duchow, Peter Harrill

**Affiliations:** 1 Internal Medicine, John F Kennedy (JFK) Medical Center/University of Miami Miller School of Medicine, Atlantis, USA; 2 Internal Medicine, John F Kennedy (JFK) Medical Center/University of Miami Miller School of Medicine, Lake Worth, USA

**Keywords:** stroke guidelines, atrial fibrillation (af), middle cerebral artery thrombosis, cerebrovascular accident (stroke), brain thrombectomy

## Abstract

Single-vessel occlusions often cause an acute ischemic stroke (AIS) but can rarely be caused by multi-vessel occlusions. Although bilateral AIS is rare, these patients often undergo mechanical thrombectomy as long as they are within the 24-hour window since symptom presentation. We present a case of a female in her 70s who presented to an outside facility with right-sided weakness in her upper and lower extremities, drooping of the right lower face, and aphasia. The patient developed bilateral symptoms on transfer to a tertiary center with neuro-interventional capabilities. Due to concern for a possible bilateral stroke, magnetic resonance imaging was ordered and was remarkable for bilateral middle cerebral artery occlusion. The patient underwent a successful bilateral mechanical thrombectomy within 24 hours of the last known normal. This case demonstrates that mechanical thrombectomy is an excellent treatment option for patients with bilateral occlusions that present within the recommended 24 hours from the last known normal.

## Introduction

Several mechanisms can cause acute ischemic stroke (AIS), and compromised vascular supply to the brain is the most common cause, making up 85%-90% of cases [1]. Only 10% of AIS is caused by multi-vessel occlusion, and it is typical for these vessels to be near one another [2]. Therefore, bilateral occlusion of the middle cerebral artery (MCA) is an extremely rare pathology that often results in poor outcomes. More specifically, patients can present with diffuse cerebral edema, brain herniation, decerebrate posturing, and death [3-6]. The treatment for such a case is often mechanical thrombectomy, given that the patient presents within six to 24 hours [7-10]. After thrombectomy, clinicians will often report the Thrombolysis in Cerebral Infarction (TICI) score, which describes the flow in the vessels after an intervention. This scoring system ranges from no perfusion (grade 0) to complete perfusion (grade 3) [11,12]. Mechanical thrombectomy, like all procedures, has a potential for complications, including puncture site hemorrhage or hematoma, embolus to a new territory, procedure-related vessel dissections, and vessel perforations leading to intracerebral hemorrhage [13,14]. We present a case of a cerebrovascular accident with a successful thrombectomy of the bilateral middle cerebral arteries.

## Case presentation

We present a case of a female in her 70s presenting to the hospital after being found with right-sided lower facial droop, aphasia, and right-sided weakness by a family member. The patient's last known normal state was 12 hours prior, at 20:30 hours, the night before. The patient's medical history was ascertained by a family member. It was significant for diabetes mellitus type 2, hypertension, dyslipidemia, and a previous cerebrovascular accident seven years prior with no residual defects. Per the family member, the patient had no deficits in sensation, motor function, or speech before the onset of symptoms. Vitals on presentation: afebrile, blood pressure of 156/67 mmHg, heart rate of 85 beats per minute, and oxygen saturation of 96% in room air. An electrocardiogram revealed sinus rhythm. The complete blood count (CBC) and basic metabolic panel (BMP) were within normal limits. The initial National Institute of Health Stroke Scale (NIHSS) score was 12 with right lower facial droop, drifting of right upper and lower extremities, and aphasia. A computerized tomography angiography (CTA) scan of the head and neck was remarkable for the occlusion of distal branches of the left middle cerebral artery. The patient was transferred to a tertiary facility for neurointervention.

**Figure 1:**
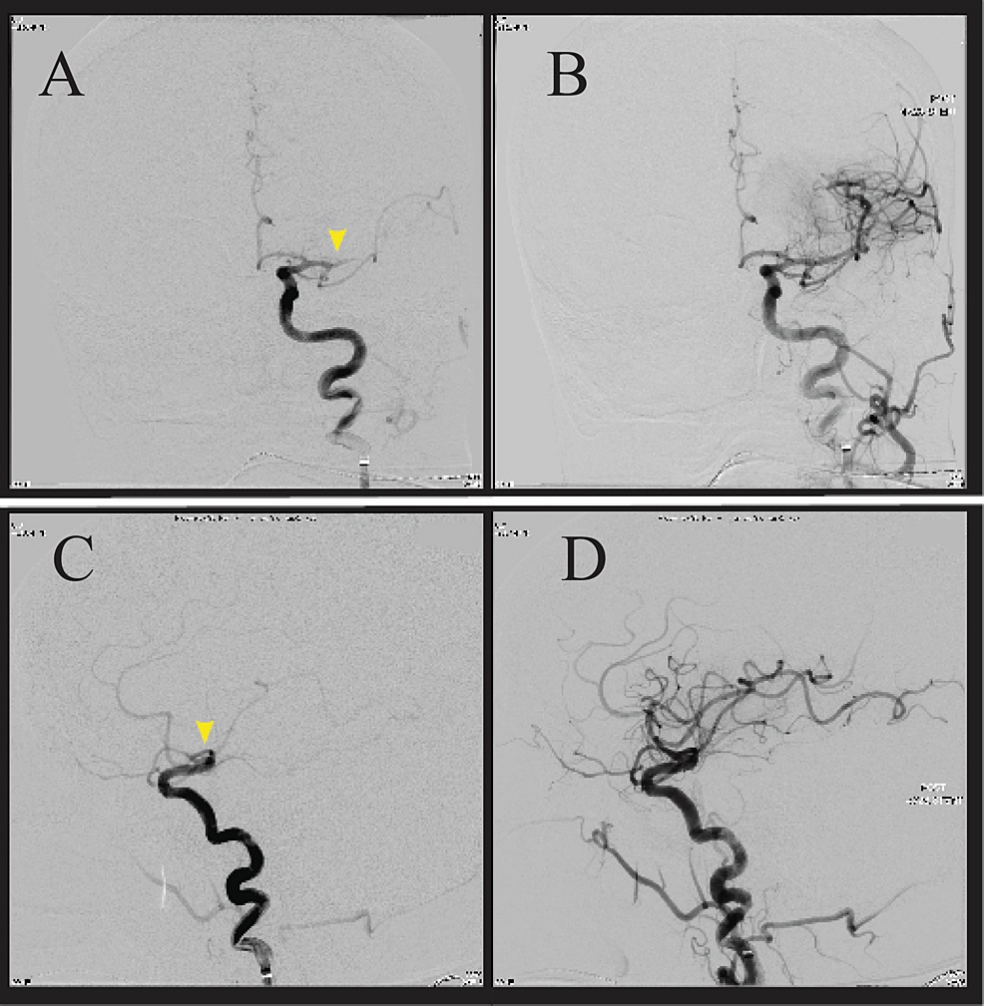
Angiogram during left MCA thrombectomy A (upper left): coronal view with M1 occlusion marked by a yellow arrow; B (upper right): coronal view with reperfusion of MCA after thrombectomy; C (lower left): sagittal view of M1 occlusion, marked by a yellow arrow; D (lower right): sagittal view with reperfusion of MCA after thrombectomy. MCA: middle cerebral artery

**Figure 2:**
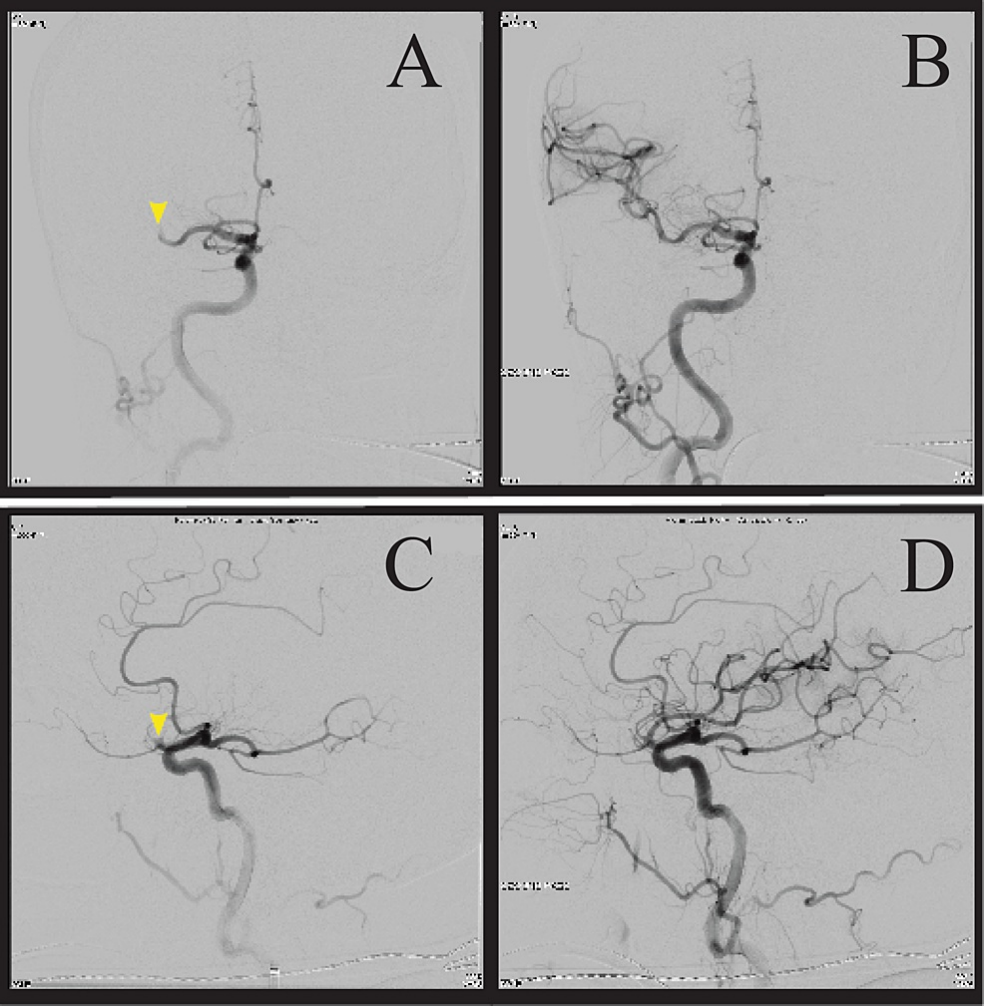
Angiogram during right MCA thrombectomy A (upper left): coronal view with M1 occlusion marked by a yellow arrow; B (upper right): coronal view with reperfusion of MCA after thrombectomy; C (lower left): sagittal view of M1 occlusion marked by a yellow arrow; D (lower right): sagittal view with reperfusion of MCA after thrombectomy MCA: middle cerebral artery

Upon arrival, the patient's vitals were significant, with a heart rate of 64 beats per minute and a blood pressure of 172/73 mmHg. She had an NIHSS score of 15 with new bilateral involvement, including drifting of the left lower extremity and being unable to perform the heel-to-shin test in both lower extremities. The patient underwent magnetic resonance imaging (MRI) at 1530 and was noted to have acute ischemia in both the right and left hemispheres within MCA territories concerning bilateral occlusion. The patient was then taken to the interventional radiology suite for a planned bilateral thrombectomy. Figure 1 shows the skin puncture at 16:35 hours with flow restoration of the left MCA at 16:58 hours and Figure 2 shows the right MCA at 17:15 hours.

The reperfusion grade for both was a TICI scale score of three.

Post-procedure, the patient remained intubated and was transferred to the neuro-ICU. On neurology review eight hours after mechanical thrombectomy, the patient had an improved NIHSS score of seven. More specifically, the patient had mild left nasolabial fold flattening, drift in the upper and lower right extremities, and minor weakness in the upper and lower left extremities. Aphasia was unable to be tested as the patient was intubated. The following day, the patient was extubated and had a remarkably improved NIHSS score of one (minor drift of the right upper extremity). A repeat CT brain scan on postoperative day (POD) two revealed a small, evolving left frontal cortical infarct and subarachnoid hemorrhage. The repeat CT brain was stable on POD three. In addition, telemetry readings while in the intensive care unit were consistent for paroxysmal atrial fibrillation, and the patient was started on therapeutic anticoagulation. The transthoracic echocardiogram was unremarkable.

Throughout the hospital course, the patient was treated with dual-antiplatelet therapy as well as a high-intensity statin with early assessment and therapy with speech and swallowing, physical therapy, and occupational therapy. The patient was discharged to home on hospital day six, with the only deficit being 4/5 strength in the right upper extremity, but no longer displayed any drift. The patient was discharged on a factor Xa inhibitor and a high-intensity statin, as well as her other home medications, with close neurology and cardiology follow-up.

## Discussion

Acute bilateral occlusion of both MCAs is exceptionally rare, usually in the setting of cardiac disease such as atrial fibrillation [2,15]. Without early intervention, this condition results in poor outcomes, including bilateral paresis, coma, and death [3-6]. The mainstay of treatment for AIS secondary to an occlusion of the internal carotid artery or the proximal MCAs is mechanical thrombectomy as long as the patient presents within six to 24 hours since the patient was last known to be well [16]. However, it has been shown that with each one-hour delay to reperfusion, the patient is at increased risk of long-term disability [17].

In this case study, our patient initially demonstrated right-sided symptoms that worsened to include bilateral involvement during transfer to a tertiary facility. As a result, the patient had further imaging done on arrival and was immediately taken to intervention once the diagnosis of bilateral M1 occlusions was made. Although the time to intervention from the last known normal was approximately 20 hours, the thrombectomy of the bilateral MCAs was successful. It resulted in complete revascularization of both occluded MCAs with a TICI score of grade 3 and an NIHSS score of 0 on discharge. This case report highlights the possible benefit of mechanical thrombectomy in bilateral MCA strokes, even within the extended window of 24 hours since the last known normal.

## Conclusions

Acute bilateral occlusion of intracerebral arteries is a rare condition that often results in poor outcomes. We presented a case of bilateral M1 occlusion in the setting of atrial fibrillation. Within 24 hours of presentation, bilateral thrombectomy was performed, leading to an excellent clinical outcome and demonstrating the effectiveness of mechanical thrombectomy in an appropriate situation.
